# Predictors of Treatment Failure in Children With HIV Starting First-line Antiretroviral Therapy in the ODYSSEY Trial

**DOI:** 10.1093/cid/ciag232

**Published:** 2026-04-10

**Authors:** James Wyncoll, Rujirek Kamolrattana, Moherndran Archary, Hilda A Mujuru, Adeodata R Kekitiinwa, Avy Violari, Sathaporn Na-Rajsima, Abbas Lugemwa, Ebrahim Variava, Mark Cotton, Cissy M Kityo, Christoph Königs, Ngundu Osee Behuhuma, Steven Welch, Yacine Saïdi, Laura Marques, Grace M Ahimbisibwe, Lily Houlden, Carlo Giaquinto, Pablo Rojo, Diana M Gibb, Thanyawee Puthanakit, Anna Turkova, Ellen White, Deborah Ford

**Affiliations:** UCL Innovative Clinical Trials Unit (formerly the MRC Clinical Trials Unit at UCL, until March 2026), Institute of Clinical Trials and Methodology, London, United Kingdom; Department of Pediatrics, Nakornping Hospital, Chiang Mai, Thailand; Department of Paediatrics, Enhancing Care Foundation, Victoria Mxenge Hospital, Durban, South Africa; Child Adolescent and Women's Health Department, University of Zimbabwe Clinical Research Centre, Harare, Zimbabwe; Department of Research, Baylor College of Medicine Children's Foundation, Kampala, Uganda; Perinatal HIV Research Unit, University of the Witwatersrand, Johannesburg, South Africa; Pediatric Department, Mahasarakam Hospital, Maha Sarakham, Thailand; Joint Clinical Research Centre (JCRC), Mbarara Regional Centre of Excellence, Mbarara, Uganda; Perinatal HIV Research Unit, University of the Witwatersrand, Johannesburg, South Africa; Department of Paediatrics and Child Health, Family Center for Research with Ubuntu, Stellenbosch University, Tygerberg, South Africa; Joint Clinical Research Centre (JCRC), Kampala, Uganda; Department of Paediatrics and Adolescent Medicine, Goethe University Frankfurt, Frankfurt, Germany; Clinical Trials Unit, Africa Health Research Institute (AHRI), Kwazulu-Natal, South Africa; Department of Paediatrics, University Hospitals Birmingham NHS Foundation Trust, Birmingham, United Kingdom; INSERM/ANRS SC10-US19, Essais Thérapeutiques et Maladies Infectieuses, Villejuif, France; Centro Materno-Infantil do Norte, ULSSA, Porto, Portugal; Makerere University-Johns Hopkins University (MU-JHU) Research Collaboration, Clinic Department, Kampala, Uganda; UCL Innovative Clinical Trials Unit (formerly the MRC Clinical Trials Unit at UCL, until March 2026), Institute of Clinical Trials and Methodology, London, United Kingdom; Department of Women and Child Health, University of Padova, Padova, Italy; Pediatric Infectious Diseases Unit, Hospital 12 de Octubre, Madrid, Spain; UCL Innovative Clinical Trials Unit (formerly the MRC Clinical Trials Unit at UCL, until March 2026), Institute of Clinical Trials and Methodology, London, United Kingdom; HIVNAT, Thai Red Cross AIDS Research Center, Bangkok, Thailand; UCL Innovative Clinical Trials Unit (formerly the MRC Clinical Trials Unit at UCL, until March 2026), Institute of Clinical Trials and Methodology, London, United Kingdom; UCL Innovative Clinical Trials Unit (formerly the MRC Clinical Trials Unit at UCL, until March 2026), Institute of Clinical Trials and Methodology, London, United Kingdom; UCL Innovative Clinical Trials Unit (formerly the MRC Clinical Trials Unit at UCL, until March 2026), Institute of Clinical Trials and Methodology, London, United Kingdom

**Keywords:** children, dolutegravir, pediatric HIV, predictors, treatment failure

## Abstract

**Background:**

Data on predictors of treatment failure in children starting antiretroviral therapy (ART) are limited, particularly on dolutegravir-based regimens (DTG).

**Methods:**

ODYSSEY demonstrated superior efficacy of DTG versus standard-of-care (SOC). We assessed predictors at ART initiation of treatment failure by 96 weeks.

**Results:**

Three hundred and eighty-one children started first-line ART (82% African). At ART-initiation, median age was 10.5 years (IQR: 6.5, 14.0, 67 < 3 years), CD4% 20% (IQR: 12, 28), BMI-for-age *Z*-score –.58 (IQR:–1.48, +.25). One hundred and eighty-nine children started DTG, 192 started SOC (91% ≥3 years started efavirenz; 79% <3 years started lopinavir). Seventy-five children experienced treatment failure (24 DTG, 51 SOC). Failure risk was lower on DTG than SOC (hazard ratio [HR] = 0.47, 95% CI: 0.29–0.77, *P* = .002). Lower BMI-for-age *Z*-score (HR = 0.82 for each unit gain, 95% CI: 0.70–0.96, *P* = .01) and being at an African site (HR = 2.09, 95% CI: 0.82–5.31, *P* = .09) were associated with higher failure risk. Risk was also higher at younger ages with the steepest increase in the youngest children and increased at lower CD4%, with a stronger CD4% effect at younger ages. At CD4% = 20, HRs relative to age 10 years were 2.40 (95% CI: 1.58–3.65) at age 1 year, 1.30 (95% CI: 1.15–1.48) at age 5 years, and 0.80 (95% CI: 0.72–0.89) at age 18 years. At age 1 year, HRs relative to CD4% = 20 were 1.39 (95% CI: 1.16–1.66) at CD4% = 15, and 0.52 (95% CI: 0.36–0.75) at CD4% = 30; at age 10, corresponding estimates were 1.07 (95% CI: 0.94–1.20) at CD4% = 15, and 0.88 (95% CI: 0.69–1.13) at CD4% = 30.

**Conclusions:**

Young age, low BMI-for-age, and low CD4% at ART initiation predicted higher risk of treatment failure and can guide targeted support.

## Teaser

ART-naïve children starting triple ART (∼50% dolutegravir-based) in the ODYSSEY trial had higher risk of treatment failure by 96 weeks if they were younger, had lower BMI-for-age or lower CD4%.

**(See the Editorial Commentary by Palumbo on pages e329–30.)**

Despite substantial progress in expanding access to antiretroviral therapy (ART) for children, treatment outcomes for children with HIV remain worse than those of adults. In 2024, 85% of children receiving ART were virologically suppressed compared with ∼94% of adults [1].

The ODYSSEY randomized trial (ClinicalTrials.gov NCT02259127) demonstrated superior 96-week efficacy of dolutegravir-based ART (DTG arm) compared with non-DTG-based standard-of-care (SOC arm) in children with HIV-1 initiating first-line or second-line ART [2, 3]. ODYSSEY data supported pediatric dolutegravir licencing and reinforced World Health Organization (WHO)'s recommendations for DTG-based ART as preferred treatment for children.

Global rollout of DTG has been rapid, with ∼85% of children on ART receiving DTG-based regimens in 2023 [4]. Several African studies have reported improved viral suppression rates in children following DTG adoption [5–8]. However, most evidence comes from ART-experienced children ≥20 kg who can take adult DTG formulations switched from alternative ART, with relatively short follow-up. Data in children <20 kg on pediatric formulations or children starting ART on DTG remain sparse [9, 10].

Studies of children on DTG, including mostly older ART-experienced children, consistently show the highest suppression rates among children suppressed prior to transition [5–8, 11]. Additional predictors of lower suppression rates include female sex [11], higher WHO stage [7], WHO-defined advanced immunosuppression [11] and poorer adherence [7]. While older age has been associated with reduced viral suppression [5], findings are inconsistent; in Mozambique, in children <9 years, the youngest did worst [10].

Retrospective cohort studies of children starting older ART regimens prior to DTG found low CD4 count, WHO stage 3/4, ongoing TB/other opportunistic infection, and death of both parents at ART initiation predicted treatment failure [12–14]. A meta-analysis of children starting first-line ART found younger age, low hemoglobin and malnutrition predicted mortality [15].

We combined the 2 ODYSSEY first-line cohorts (children enrolled weighing ≥14 kg and <14 kg), half started on DTG-based regimens, in an exploratory analysis of predictors of treatment failure.

## METHODS

ODYSSEY recruited 707 children weighing ≥14 kg and <18 years of age between September 2016 and June 2018, including 311 who started first-line ART (309 with follow-up) [2]. After dolutegravir dispersible tablets became available for younger children, an additional 85 children weighing 3 to <14 kg, aged at least 28 days, were recruited between July 2018 and August 2019, including 72 who started first-line ART [3]. The main exclusion criteria were severe liver disease, pregnancy, and breast-feeding.

Information collected prior to/at enrollment included physical measurements, laboratory measurements, HIV disease staging, and demographics. In follow-up, participants were assessed at weeks 2 (<14 kg cohort), 4, 12, 24, and every 12 weeks thereafter.

The trial's primary endpoint was treatment failure by 96 weeks, defined as the first occurrence of: insufficient virological response (viral load decline <1 log10 by week 24, or viral load ≥50 copies/mL at week 24 if baseline viral load was <500 copies/mL, followed by ART switch for treatment failure); virologic failure (2 consecutive viral loads ≥400 copies/mL, with the first at or after week 36); a new or recurrent WHO stage 4 or severe WHO stage 3 event; or death from any cause.

Baseline predictors of treatment failure by 96 weeks were investigated using Cox regression. Time was measured from randomization and censored at loss to follow-up, study withdrawal, or 96 weeks. Fourteen baseline characteristics were grouped into 4 domains (anthropometrics: weight, middle-upper arm circumference [MUAC], height-for-age and BMI-for-age; HIV clinical indicators: CD4%, CD4/CD8 ratio, viral load and WHO stage; hematology: neutrophils, hemoglobin and platelets; demographics: sex, primary caregiver and region). CD4 results were expressed as percentages of total lymphocyte counts, which vary less with age [16]. Height- and BMI-for-age *Z*-scores were derived using WHO Child Growth Standards [17]. Weight-for-age was excluded because reference standards apply only to children <10 years and, in children <10 years, weight-for-age and BMI-for-age were strongly correlated. Region of clinical site was classified as African or non-African. Sex of primary caregiver was considered.

There were few missing data, with complete data for 8 of 16 variables including trial arm and age. For primary caregiver, the most recent information provided prior to randomization was used; if unavailable, the next form was used, provided it was within 4 weeks post randomization. Data on 12% of caregivers were missing and 4% could not be categorized by sex due to non-sex-specific relation (eg cousin). For continuous variables with missing data (≤4% for all, except neutrophils at 6%), mean imputation was used in multivariable models.

Continuous variables, for which a monotonic relationship with risk was clinically appropriate and supported by the data, were modeled using first-degree fractional polynomials (with powers −2, −1, −0.5, 0.5, 1, and 2) to preserve statistical power [18]. The best nonlinear first-degree fractional polynomial form was selected over a linear model using a likelihood ratio test (*P* < .05). Although the best nonlinear form for age (log_e_) was not statistically superior to linear age in a model adjusted for treatment arm (*P* = .14), we used log_e_(age) in all models to reflect the steeper decline in risk at younger ages. Log viral load was modeled categorically due to fluctuation in risk with increasing viral load.

Predictors were evaluated in “univariable” models adjusted for trial arm and adjusted for trial arm and age. “Multivariable” models included the following: “domain-specific models” containing variables within the same domain; and a “full multivariable model” including variables across domains, all adjusted for trial arm and age. Interactions between predictors and trial arm were assessed in all multivariable models.

Within domain, where pairwise collinearity was detected (correlation coefficient r ≥ 0.80), the variable yielding the better model fit after adjustment for trial arm and age (based on Akaike Information Criterion, AIC) was retained. Remaining variables were entered into domain-specific multivariable models and selected using backward elimination (retention threshold *P* < .2, using likelihood ratio tests). Variables retained across domains were then considered in a full multivariable model, using backward elimination (*P* < .1). Effect modification by age was examined by adding interaction terms to the final model. Factors from the final model were fitted separately within trial arm and within age-group (<3 years/≥3 years).

To assess the robustness of selected prognostic factors to sampling variability, bootstrap resampling (1000 samples) was applied to domain-specific models and the full multivariable model [19]. For bootstrapping the full multivariable model, candidate variables were those retained by significance testing in the domain-specific models. Bootstrap models included trial arm and log_e_(age) and tested other factors independently (with no interactions).

An alternative definition of treatment failure was considered raising the threshold for virological failure to confirmed viral load ≥1000 copies/mL, keeping other failure criteria unchanged.

## RESULTS

Of 383 children who initiated first-line ART, 381 were included (2 SOC participants had no follow-up). Overall, 52% were female, and 82% were African. At ART initiation, median age was 10.5 years (IQR: 6.5–14.0); 314 ≥ 3 years and 67 < 3 years, aligning closely with weight cohorts (Supplementary Appendix 1). Median CD4% was 20% (IQR: 12–28) and median BMI-for-age *Z*-score was −0.58 (IQR:−1.48 to +0.25). Seventy-three (19%) children had at least one ongoing WHO stage 3/4 event, including 40 with tuberculosis and 20 with malnutrition. One hundred and eighty-nine children were assigned to DTG and 192 to SOC; among SOC, 91% of those ≥3 years started efavirenz and 79% of those <3 years started boosted lopinavir (12% efavirenz, 9% nevirapine). Most children (89%; 309/348) acquired HIV perinatally. Baseline characteristics were well balanced between trial arms (Table 1, Supplementary Appendix 1).

**Table 1.** Baseline Characteristics and Predictors of Treatment Failure

	Total (% of Total)	Failed (% of Subgroup)	“Univariable” Model, Adjusted for Trial Arm			“Univariable” Model Adjusted for Trial Arm and Baseline Age		
Factor			HR	95% CI	*P* Value	HR	95% CI	*P* Value
Total	381 (100%)	75 (20%)						
Trial arm (SOC)	192 (50%)	51 (27%)	1.00			1.00		
DTG	189 (50%)	24 (13%)	0.44	(.27–.72)	<.001	0.44	(.27–.72)	<.001
Age—per year increase (median [IQR])	10.5 [6.5–14.0]		^a^	^a^	<.001	^a^	^a^	<.001
4 wks to < 3 y	67 (18%)	25 (37%)	1.00		<.001			
3 y to < 6 y	17 (4%)	4 (24%)	0.57	(.20–1.64)				
6 to < 12 y	154 (40%)	29 (19%)	0.45	(.26–.77)				
12 to < 18 y	143 (38%)	17 (12%)	0.28	(.15–.53)				
Weight—per kilo increase (median [IQR]) ^b^	26.4 [18.4–41.0]		^c^	^c^	<.001			
3 to < 6 kg	23 (6%)	13 (57%)	1.00		<.001			
6 to < 10 kg	35 (9%)	11 (31%)	0.45	(.20–1.01)				
10 to < 14 kg	14 (4%)	2 (14%)	0.19	(.04–.86)				
14 to < 20 kg	38 (10%)	10 (26%)	0.35	(.15–.81)				
20 to < 40 kg	173 (45%)	30 (17%)	0.24	(.13–.46)				
≥40 kg	98 (26%)	9 (9%)	0.12	(.05–.28)				
Middle-upper arm circumference—per cm increase (median [IQR]) (n = 372)	18.0 [15.5–21.7]		^d^	^d^	<.001			
<15	72 (19%)	29 (40%)	1.00		<.001			
15 to < 18	107 (29%)	23 (22%)	0.46	(.27–.80)				
18 to < 21	79 (21%)	11 (14%)	0.29	(.15–.59)				
≥21	114 (31%)	12 (11%)	0.21	(.11–.42)				
Height-for-age z-score—per unit increase (median [IQR])	−1.3 [−2.3 to −.5]		0.74	(.63–.88)	<.001	0.83	(.70–.98)	.03
<−3	52 (14%)	19 (37%)	1.00		.001	1.00		.09
−3 to < −2	64 (17%)	16 (25%)	0.55	(.28–1.07)		0.74	(.37–1.49)	
−2 to < 0	221 (58%)	34 (15%)	0.33	(.19–.58)		0.50	(.27–.94)	
≥0	44 (12%)	6 (14%)	0.26	(.10–.65)		0.37	(.14–.97)	
BMI-for-age *z*-score—per unit increase (median [IQR])	−0.58 [−1.48–0.25]		0.70	(.60–.82)	<.001	0.75	(.64–.87)	<.001
<−3	21 (6%)	13 (62%)	1.00		<.001	1.00		<.001
−3 to < −2	35 (9%)	11 (31%)	0.29	(.13–.66)		0.35	(.15–.78)	
−2 to < 0	205 (54%)	33 (16%)	0.16	(.08–.30)		0.21	(.11–.40)	
≥0	120 (32%)	18 (15%)	0.15	(.07–.30)		0.19	(.09–.40)	
CD4%—per % increase (median [IQR]) (n = 377)	20 [12–28]		0.98	(.96–1.00)	.02	0.96	(.94–.98)	<.001
<15%	125 (33%)	31 (25%)	1.00		.06	1.00		.004
15 to < 25%	126 (33%)	25 (20%)	0.78	(.46–1.32)		0.58	(.33–1.01)	
≥25%	126 (33%)	17 (13%)	0.49	(.27–.89)		0.36	(.19–.66)	
CD4/CD8 ratio—per 0.1 increase (median [IQR]) (n = 369)	0.4 [0.2–0.7]		0.95	(.89–1.01)	.07	0.92	(0.86–0.98)	.002
<0.2	89 (23%)	26 (29%)	1.00		.08	1.00		.004
0.2 to < 0.6	165 (43%)	28 (17%)	0.57	(.33–.97)		0.46	(.27–.80)	
0.6 to < 1.0	78 (20%)	12 (15%)	0.44	(.22–.87)		0.33	(.16–.66)	
≥1.0	49 (13%)	9 (18%)	0.59	(.28–1.25)		0.30	(.13–.69)	
Viral load—copies/mL								
<1000	29 (8%)	5 (17%)	1.00		.02	1.00		.20
1000 to < 10 000	66 (18%)	10 (15%)	0.82	(.28–2.39)		0.91	(.31–2.69)	
10 000 to < 50 000	85 (23%)	13 (15%)	0.89	(.32–2.51)		0.94	(.34–2.65)	
50 000 to < 100 000	47 (13%)	4 (9%)	0.47	(.13–1.75)		0.51	(.14–1.89)	
100 000 to < 1 000 000	129 (34%)	36 (28%)	1.75	(.69–4.45)		1.55	(.61–3.96)	
≥1 000 000	19 (5%)	5 (26%)	1.91	(.55–6.60)		1.07	(.30–3.85)	
(median [IQR]—log10) (n = 375)	4.7 [4.0–5.3]							
<100 000	227 (61%)	32 (14%)	1.00			1.00		
≥100 000	148 (39%)	41 (28%)	2.22	(1.40–3.53)	<.001	1.76	(1.08–2.87)	.02
Ongoing WHO stage 3/4 event (no)	308 (81%)	50 (16%)	1.00			1.00		
Yes	73 (19%)	25 (34%)	2.49	(1.54–4.03)	<.001	2.29	(1.42–3.71)	.001
Hemoglobin—per 1 unit increase (g/dL) (median [IQR]) (n = 379)	11.2 [10.0–12.5]		0.84	(.75–.94)	.002	0.91	(.80–1.03)	.14
<10	89 (23%)	24 (27%)	1.00		.002	1.00		.11
10 to < 12	162 (43%)	36 (22%)	0.73	(.43–1.22)		0.93	(.54–1.60)	
≥12	128 (34%)	13 (10%)	0.32	(.16–0.63)		0.49	(.23–1.05)	
Platelets—per 10 units increase (10^3^/µL) (median [IQR]) (n = 379)	325 [252–407]		^e^	^e^	.04	^e^	^e^	.12
<250	91 (24%)	22 (24%)	1.00		.15	1.00		.19
250 to < 325	97 (26%)	16 (16%)	0.63	(.33–1.20)		0.67	(.35–1.28)	
325 to < 400	90 (24%)	12 (13%)	0.54	(.27–1.10)		0.49	(.24–0.99)	
≥400	101 (27%)	23 (23%)	1.01	(.56–1.82)		0.84	(.46–1.53)	
Neutrophils—per 1 unit increase (10^9^/L) (median [IQR]) (n = 358)	2.1 [1.5–3.0]		1.21	(1.08–1.36)	.004	1.17	(1.05–1.32)	.01
<1	29 (8%)	5 (17%)	1.00		.07	1.00		.07
1 to < 2	140 (39%)	24 (17%)	1.11	(.42–2.91)		1.26	(.48–3.32)	
2 to < 3	96 (27%)	15 (16%)	0.96	(.35–2.64)		0.99	(.36–2.72)	
≥3	93 (26%)	27 (29%)	2.01	(.77–5.22)		2.13	(.82–5.55)	
Sex—male	181 (48%)	39 (22%)	1.00			1.00		
Female	200 (52%)	36 (18%)	0.87	(.55–1.37)	.56	0.95	(.60–1.51)	.84
Primary caregiver—female (n = 321)	270 (84%)	59 (22%)	1.00			1.00		
Male	51 (16%)	6 (12%)	0.51	(.22–1.18)	.08	0.66	(.28–1.56)	.32
Region (non-African site)	68 (18%)	5 (7%)	1.00			1.00		
African site^f^	313 (82%)	70 (22%)	3.08	(1.24–7.63)	.004	2.20	(.87–5.59)	.07

Data are complete (381 observations) unless otherwise stated. All *P*-values are from likelihood ratio tests. Variables were transformed using the best-fitting first-degree fractional polynomial. Weight and MUAC were collinear with baseline age; therefore, “univariable” models adjusted for trial arm and age for these variables are not presented in the table. Where >1 category, global *P*-values are shown.

Abbreviations: BMI, body mass index; CI, confidence interval; DTG, dolutegravir; HR, hazard ratio; IQR, interquartile range; SOC, standard of care; WHO, World Health Organization.

^a^Age was modeled as log_e_(age); Supplementary Appendix 2A.

^b^Median [IQR] age for weight cohorts: <14 kg cohort: 1.1 [0.5–1.9], ≥14 kg cohort: 11.8 [8.8–14.9].

^c^Weight was transformed using 1/√weight. The HR for risk of outcome in a child weighing 14 kg versus 4 kg was 0.27 (95% CI: 0.17–0.42), and for 24 kg versus 14 kg was 0.70 (0.62–0.79).

^d^MUAC was transformed using 1/MUAC^2^. The HR comparing 15 cm versus 10 cm was 0.18 (95% CI: 0.11–0.30), and 25 cm versus 20 cm was 0.76 (0.70–0.82).

^e^Platelets were transformed using 1/√platelets. The HR for 100 × 10^3^/µL versus 50 × 10^3^/µL was 0.75 (95% CI: 0.60–0.94), and for 350 × 10^3^/µL versus 300 × 10^3^/µL was 0.97 (0.95–0.99) adjusted for trial arm only.

^f^34% of total participants were from Uganda, 25% South Africa, 23% Zimbabwe (13% Thailand, 5% Europe).

Most participants (369 [97%]) were seen at or after 96 weeks or met the primary endpoint. By 96 weeks, 75 (20%) participants experienced treatment failure (24 DTG, 51 SOC): 56 (75%) due to virological failure, 2 (3%) due to insufficient virological response, 11 (15%) due to a new or recurrent WHO Stage 4 event, and 6 (8%) due to death.

Adjusting for trial arm only, the strongest predictors of treatment failure were age, weight, MUAC, height-for-age, BMI-for-age, viral load, and ongoing WHO stage 3/4 event at ART initiation (all *P* < .001; Table 1). There were weaker effects for CD4%, CD4/CD8 ratio, hemoglobin, platelets, neutrophils, primary caregiver, and region (*P* < .1). After additionally adjusting for age, primary caregiver was no longer predictive of treatment failure (*P* ≥ .2), while CD4% became a stronger predictor (*P* < .001).

### Baseline Age and Allocated ART Regimen as Predictors of Treatment Failure

In a joint model including trial arm and age, the risk of treatment failure was lower in the DTG arm than SOC (HR = 0.44, 95% CI: 0.27–0.72, *P* < .001; Table 1). Risk was strongly associated with age (*P* < .001) and was higher at younger ages with the steepest increase in the youngest children (*P* = .14 for non-linearity; Table 1, Supplementary Appendix 2).

### Anthropometric Measures as Predictors of Treatment Failure

Weight and MUAC were collinear with log_e_(age) (r = 0.93, *P* < .001; r = 0.81, *P* < .001, respectively) and were not considered in multivariable models. Lower BMI-for-age independently predicted treatment failure (HR = 0.74 per unit increase, 95% CI: 0.63–0.86, *P* < .001; Table 2). Height-for-age was not retained (*P* = .21). Bootstrap resampling strongly supported including BMI-for-age (selected in 97% of samples) and showed moderate support for height-for-age (43%, Supplementary Appendix 3).

**Table 2.** Multivariable Predictors of Treatment Failure

	Anthropometrics			HIV Clinical Indicators			Hematology			Demographics			Full Multivariable Model		
Factor	HR	95% CI	*P*Value	HR	95% CI	*P*Value	HR	95% CI	*P*Value	HR	95% CI	*P*Value	HR	95% CI	*P*Value
Trial arm (DTG)	0.46	(.28–.75)	.002	0.45	(.28–.73)	.001	0.46	(.28–.75)	.002	0.45	(.27–.72)	<.001	0.46	(.28–.74)	.001
Age—per year increase	^a^	^a^	<.001	^a^	^a^	<.001	^a^	^a^	<.001	^a^	^a^	.001	^a^	^a^	<.001
BMI-for-age—per unit increase	0.74	(.63–.86)	<.001	…	…		…	…		…	…		0.81	(.69–.95)	.01
Height-for-age—per unit increase	-	-	-	…	…		…	…		…	…		…	…	
CD4%—per % increase	…	…		0.97	(.95–.99)	.005	…	…		…	…		.97	(.95–1.00)	.02
Viral load (≥100 000 copies/mL)	…	…		-	-	-	…	…		…	…		…	…	
Ongoing WHO 3/4 event (yes)	…	…		1.92	(1.17–3.16)	.01	…	…		…	…		-	-	-
Neutrophils—per unit increase (10^9^/L)	…	…		…	…		1.17	(1.04–1.31)	0.01	…	…		-	-	-
Hemoglobin—per 1 unit increase (g/dL)	…	…		…	…		-	-	-	…	…		…	…	
Platelets—per 10 units increase (10^3^/µL)	…	…		…	…		-	-	-	…	…		…	…	
Region (African site)	…	…		…	…		…	…		2.20	(.87–5.59)	.07	2.22	(0.87–5.64)	.06
Sex (female)	…	…		…	…		…	…		…	…		…	…	

... denote variables that were not considered for the model. − denote variables that were considered but not retained in the model selection procedure (backwards elimination (*P* < .1)). All *P* values are from likelihood ratio tests.

Missing values for CD4%, viral load, neutrophils, hemoglobin, and platelets were imputed using mean imputation.

Abbreviations: BMI, body mass index; CI, confidence interval; DTG, dolutegravir; HIV, human immunodeficiency virus; HR, hazard ratio; WHO, World Health Organization.

^a^Age was transformed using log(age); Supplementary Appendix 2B.

### HIV Clinical Indicators as Predictors of Treatment Failure

CD4/CD8 ratio was collinear with CD4% (r = 0.81; *P* < .001). Since CD4% demonstrated a better model fit (AIC difference = −13), it was considered in preference to CD4/CD8 ratio. Lower CD4% (HR = 0.97 per percent gain, 95% CI: 0.95–0.99, *P* = .005; Table 2) and an ongoing WHO stage 3/4 event (HR = 1.92, 95% CI: 1.17–3.16, *P* = .01) independently predicted treatment failure. History of a prior WHO stage 3/4 event, if there was no ongoing WHO stage 3/4 event at ART initiation, was not an additional predictor of treatment failure (*P* = .47). High baseline viral load (≥100 000 copies/mL) was not retained (*P* = .33) after adjustment for CD4% and an ongoing WHO stage 3/4 event. Bootstrap resampling strongly supported including CD4% (selected in 84% of samples) and ongoing WHO stage 3/4 event (75% of samples). Viral load was retained in only 25% of samples (Supplementary Appendix 3).

### Hematological Predictors of Treatment Failure

Increasing neutrophil count was independently associated with increased risk of treatment failure (HR = 1.17 per cell/mm^3^ increase, 95% CI: 1.04–1.31, *P* = .01; Table 2). Hemoglobin (*P* = .27) and platelets (*P* = .24) were not retained. Bootstrap resampling strongly supported including neutrophil count (selected in 82% of samples), and there was moderate support for hemoglobin (44%) and platelets (48%, Supplementary Appendix 3).

### Demographic Predictors of Treatment Failure

Since sex of primary caregiver was missing for some participants and was not associated with treatment failure after adjustment for trial arm and age (*P* = .32; Table 1), we considered a domain-specific model including region and participant sex. Being at an African site independently predicted treatment failure (HR = 2.20, 95% CI: 0.87–5.59, *P* = .07; Table 2). Sex was not retained (*P* = .68). Bootstrap resampling gave strong support for region (selected in 76% of samples) and limited support for sex (23%, Supplementary Appendix 3).

### Overall Predictors of Treatment Failure

Trial arm, age, BMI-for-age, CD4%, and region independently predicted treatment failure (Table 2). The effect of CD4% differed by log_e_(age) (*P* = .009), so an interaction term between log_e_(age) and CD4% was included in the final model. No other predictors showed evidence of age-related effect modification (*P* > .1).

Failure risk remained consistently lower on DTG than SOC (HR = 0.47, 95% CI: [0.29–0.77], *P* = .002; Table 3). Lower BMI-for-age *Z*-score (HR = 0.82 for each unit gain, 95% CI: 0.70–0.96, *P* = .01) and being at an African site (HR = 2.09, 95% CI: 0.82–5.31, *P* = .09) were also independently associated with higher failure risk.

**Table 3.** Final Multivariable Model

Factor	Comparison	Assumption	HR	95% CI	*P* Value
Trial arm	DTG versus SOC		0.47	(.29–.77)	.002
Age (y)	1 versus 10	CD4% = 20%	2.40	(1.58–3.65)	<.001^a^
	2 versus 10	CD4% = 20%	1.84	(1.38–2.47)	
	5 versus 10	CD4% = 20%	1.30	(1.15–1.48)	
	18 versus 10	CD4% = 20%	0.80	(.72–.89)	
BMI-for-age	Per unit increase		0.82	(.70–.96)	.01
CD4%	15% versus 20%	Age = 1	1.39	(1.16–1.66)	.002^a^
	30% versus 20%	Age = 1	0.52	(.36–.75)	
	15% versus 20%	Age = 10	1.07	(.94–1.20)	
	30% versus 20%	Age = 10	0.88	(.69–1.13)	
Region (African site)	African site versus non-African site		2.09	(.82–5.31)	.09

^a^For age and CD4%, *P* values were obtained from joint likelihood ratio tests of the main effect and the age × CD4% interaction term. Age × CD4% interaction *P* = .008.

Abbreviations: BMI, body mass index; CI, confidence interval; DTG, dolutegravir; HR, hazard ratio.

Risk was higher at younger ages with the steepest increase in the youngest children. At a CD4%=20%, hazard ratios (HRs) relative to age 10 years were 2.40 (95% CI: 1.58–3.65) at age 1 year, 1.84 (95% CI: 1.38–2.47) at age 2 years, 1.30 (95% CI: 1.15–1.48) at age 5 years, and 0.80 (95% CI: 0.72–0.89) at age 18 years. Risk increased at lower CD4%, with a stronger effect of CD4% at younger ages. At 1 year, HRs relative to CD4%=20% were 1.39 (95% CI: 1.16–1.66) at CD4%=15%, and 0.52 (95% CI: 0.36–0.75) at CD4%=30%. While at age 10, corresponding associations were weaker (HR = 1.07 [95% CI: 0.94–1.20] at CD4%=15%; HR = 0.88 [95% CI: 0.69–1.13] at CD4%=30%; Table 3, Supplementary Appendix 4).

Ongoing WHO stage 3/4 event and neutrophils were not retained after adjusting for BMI-for-age. BMI-for-age (76%), CD4% (64%), and region (58%) were most frequently selected in bootstrap samples; ongoing WHO stage 3/4 event (52%) and neutrophils (50%) were selected at moderate frequencies (Supplementary Appendix 3). There was no evidence that predictors differed between trial arms, and models fitted within trial arm showed similar associations (all interactions *P* > .1, Supplementary Appendix 5). When region was excluded from model selection, the multivariable model included the same factors as the final model (Supplementary Appendix 6).

**Figure 1.**
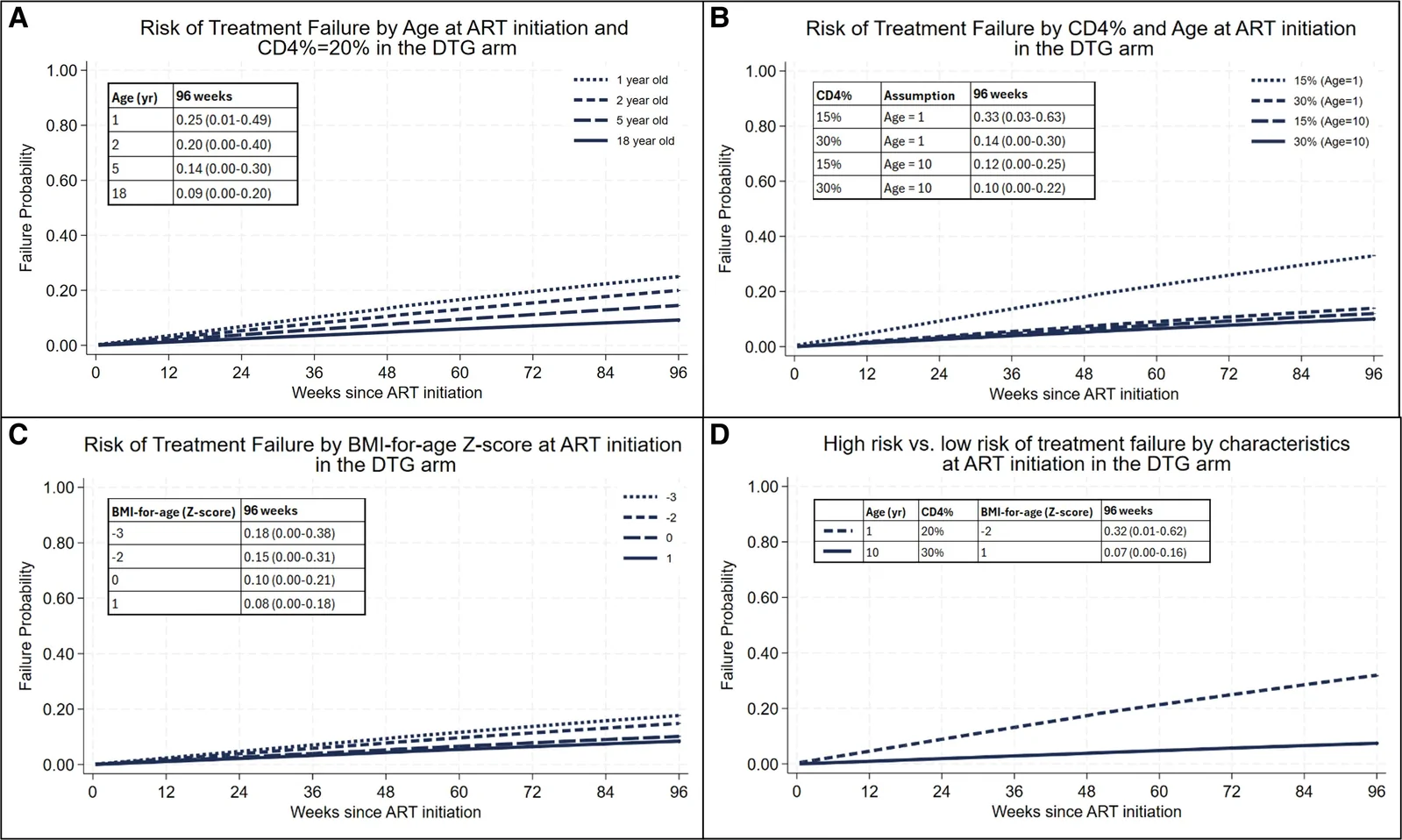
Risk of treatment failure by age, body mass index (BMI)-for-age and CD4% at antiretroviral therapy (ART) initiation in the dolutegravir (DTG) arm. Risks are predicted from the full multivariable model and shown (*A*) by age at ART initiation (1, 2, 5, and 18 y), with CD4% and BMI-for-age *Z*-score fixed at their medians (20% and −0.58 *Z*-score, respectively); (*B*) as combinations by CD4% at ART initiation (15% and 30%) and age at ART initiation (1 y and 10 y) with BMI-for-age *Z*-score fixed at its median (−0.58 *Z*-score); (*C*) by BMI-for-age *Z*-score at ART initiation (−3, −2, 0, and 1), with age fixed at 10 y and CD4% fixed at its median (20%). *D,*comparison of predicted high-risk (Age = 1, CD4% = 20%, BMI-for-age *Z*-score = −2) versus low-risk (Age = 10, CD4% = 30%, BMI-for-age *Z*-score = 1) profiles based on baseline characteristics, with all predictors set as specified. All predictions assume treatment with DTG at an African study site. Results are presented as probabilities of treatment failure by 96 wks, with 95% CIs. Abbreviations: ART, antiretroviral therapy; BMI, body mass index; CI, confidence interval; DTG, dolutegravir.

Figure 1, Table 4, and Supplementary Appendix 7 show model-based estimated risks of treatment failure for different baseline characteristics.

**Table 4.** Predicted Probabilities of Treatment Failure by 96 Weeks by Age, BMI-for-Age, and CD4% at ART Initiation in the DTG Arm at an African Site

	BMI-for-Age Z-score					
	−2			0		
	CD4%			CD4%		
	15%	20%	30%	15%	20%	30%
Age (y)	Risk (95% CI)	Risk (95% CI)	Risk (95% CI)	Risk (95% CI)	Risk (95% CI)	Risk (95% CI)
1	0.41 (0.05–0.77)	0.32 (0.01–0.62)	0.18 (0.00–0.39)	0.30 (0.02–0.58)	0.23 (0.00–0.45)	0.13 (0.00–0.27)
2	0.31 (0.02–0.61)	0.25 (0.00–0.51)	0.16 (0.00–0.35)	0.22 (0.01–0.44)	0.18 (0.00–0.36)	0.11 (0.00–0.24)
5	0.21 (0.00–0.43)	0.19 (0.00–0.39)	0.14 (0.00–0.32)	0.15 (0.00–0.30)	0.13 (0.00–0.27)	0.10 (0.00–0.21)
10	0.16 (0.00–0.33)	0.15 (0.00–0.31)	0.13 (0.00–0.30)	0.11 (0.00–0.22)	0.10 (0.00–0.21)	0.09 (0.00–0.20)
18	0.12 (0.00–0.25)	0.12 (0.00–0.26)	0.12 (0.00–0.28)	0.08 (0.00–0.17)	0.08 (0.00–0.17)	0.08 (0.00–0.19)

Abbreviations: BMI, body mass index; CI, confidence interval; DTG, dolutegravir.

### Impact of Revising the Definition of Virological Failure on Model Selection

Of the 56 participants who met the primary endpoint based on confirmed viral load ≥400 copies/mL, 47 (84%) also had confirmed viral load ≥1000 copies/mL by 96 weeks. Using the higher threshold for virological failure within the composite endpoint, the selected full multivariable model included trial arm, age, height-for-age, CD4% and an ongoing WHO stage 3/4 event. The final multivariable model under the original definition also showed good model fit under the revised definition (AIC difference = 3, Supplementary Appendix 8).

### Alternative Model Selections

When predictors were considered separately in children <3 years and ≥3 years, the stronger effects of age and CD4% in children <3 years were apparent, whereas viral load ≥100 000 copies/mL appeared more predictive of failure than CD4% in the older children (Supplementary Appendix 9).

In models reflecting limited laboratory availability, viral load was predictive when CD4% was unavailable, and an ongoing WHO stage 3/4 event was predictive when both CD4% and viral load were unavailable (Supplementary Appendix 10).

## DISCUSSION

This study adds to the evidence on predictors of treatment failure identifiable in children at initiation of first-line DTG-based ART, including some of the first robust data on younger children starting DTG <20 kg. At ART initiation, younger age, lower BMI-for-age, lower CD4%, presence of a WHO stage 3/4 event, and elevated neutrophil count predicted treatment failure. ODYSSEY included ∼50% of children starting DTG-regimens, while in the SOC arm, older children mostly started efavirenz and younger children lopinavir/ritonavir. Importantly, we found no evidence that predictors differed between DTG and SOC and DTG was consistently associated with an approximately 50% lower failure risk across subgroups with different baseline characteristics. These predictors had mostly been described in studies of children on older ART regimens, reinforcing our conclusion that the same predictors identify high-risk children on DTG.

At ART initiation, age strongly predicted treatment failure, with younger age associated with increasing risk, and a steeper increase in risk among the youngest children. The EARTH cohort, reported high mortality in the first 2 years of life (12%) and poor virological control, despite very early ART [20], likely reflecting adherence challenges, limitations of older regimens or prior exposure to perinatal antiretroviral therapy. Our findings show that even with optimized pediatric formulations, treatment failure remains high in the youngest children. EARTH also highlighted the association of maternal social environment on outcomes in young children. In ODYSSEY, deaths and treatment failure were disproportionately higher among children starting ART before 1 year (9% mortality; 38% treatment failure) compared with those ≥1 year (1% mortality, 16% failure). While some studies have shown higher risks of treatment failure in adolescents on DTG-based ART, they included few very young children, comparing adolescents with children mostly ≥5 years or older [5, 7, 11]; these findings may also reflect poorer “real-world” clinic attendance and adherence in adolescents [21–23].

Our findings align with existing literature demonstrating the adverse impact of malnutrition on HIV treatment outcomes [24, 25]. The association between low BMI-for-age and treatment failure likely reflects several interconnected biological mechanisms. Malnutrition significantly compromises immune function in children with HIV by increasing microbial translocation, immune activation, and immune cell exhaustion. An observational study in children aged 2 months to 12 years in South Africa found that malnutrition was independently associated with reduced CD4% immune recovery after 48 weeks of ART, providing a plausible explanation for our findings [26]. Malnutrition has also been linked to accelerated HIV disease progression [27] and an increased risk of developing tuberculosis [28].

For HIV clinical indicators, low CD4% and an ongoing WHO stage 3/4 event were identified as predictors of treatment failure, findings which are supported by previous research [5, 11, 13, 29, 30]. This may be explained by the fact that children with low CD4 counts, and advanced HIV are more susceptible to opportunistic infections, which can complicate their treatment response [13].

Underlying coinfections may explain our finding that a higher neutrophil count was associated with treatment failure in this study [31]. Elevated neutrophil counts are often higher in individuals with concurrent infections and inflammatory conditions [32]. Chronic inflammation in HIV is associated with sustained neutrophil activation, driven by the basal release of proinflammatory cytokines, including IL-18, IL-22, TGF-B, and IL-8 [33].

Strengths of our study include the wide age-range (with 21% < 5 years at ART initiation), the inclusion of participants from 3 African countries, Europe, and Thailand, comparative data on DTG-based ART and other regimens and very low loss to follow-up with minimal missing data. However, the use of clinical trial data may limit generalizability, as participants received frequent follow-up at established sites, likely lowering observed failure rates, and possibly contributing to the excellent retention and adherence in adolescents. Older children starting ART are often slow or long-term nonprogressors and late presenters. These groups differ in their risk of treatment failure compared with the broader pediatric population with perinatally acquired HIV. Therefore, the composition of the ODYSSEY population is likely to have impacted the estimated age effect, and this should be considered when generalizing our findings. Further research using real-world data following the global implementation of DTG-based regimens is needed to validate these findings. We found being at an African site was associated with higher risk of treatment failure, which likely reflects unidentified predictors. For example, information on carers’ characteristics, social and household factors were not collected, and these may be particularly important for predicting risk of treatment failure in young children.

Our findings demonstrate that even on DTG-based ART, some children remain at high risk of treatment failure and require additional support. They enforce the need for strong, sustained support for mothers during pregnancy and early life of infants to improve outcomes in the youngest children. Screening for and prevention of opportunistic infections such as tuberculosis should be routine in children starting ART and nutritional support should be considered. Baseline CD4% (preferably by point-of-care) would help identify those at the highest risk of treatment failure and should be retained for ART initiation assessments. Future research should evaluate interventions, such as adherence support, non-oral long-acting ART and enhanced treatments, for high-risk children.

## Supplementary Material

ciag232_Supplementary_Data

## Supplementary Data

Supplementary materials are available at *Clinical Infectious Diseases* online. Consisting of data provided by the authors to benefit the reader, the posted materials are not copyedited and are the sole responsibility of the authors, so questions or comments should be addressed to the corresponding author.
