# Supplementary material for: Predictors of Treatment Failure in Children With HIV Starting First-line Antiretroviral Therapy in the ODYSSEY Trial
Source: Clin Infect Dis. 2026 Apr 10;83(2):e320–8. doi: 10.1093/cid/ciag232 (PMC13537162; doi:10.1093/cid/ciag232)
Supplement: Supplementary file 1 [file ciag232_supplementary_data.docx]

**Appendix 1:** Baseline characteristics by age group

|  | **Total** | | **<3yrs** | | **>=3yrs** | |
| --- | --- | --- | --- | --- | --- | --- |
|  | **Total (% of Total)** | **Failed (% of Subgroup)** | **Total (% of Total)** | **Failed (% of Subgroup)** | **Total (% of Total)** | **Failed (% of Subgroup)** |
| **Factor** |  |  |  |  |  |  |
| **Total** | 381 (100%) | 75 (20%) | 67 (100%) | 25 (37%) | 314 (100%) | 50 (16%) |
| **Trial Arm (DTG) *** | 189 (50%) | 24 (13%) | 33 (49%) | 9 (27%) | 156 (50%) | 15 (10%) |
| SOC | 192 (50%) | 51 (27%) | 34 (51%) | 16 (47%) | 158 (50%) | 35 (22%) |
| EFV | 147 (77%) | 34 (23%) | 4 (12%) | 1 (25%) | 143 (91%) | 33 (23%) |
| LOP | 32 (17%) | 14 (44%) | 27 (79%) | 13 (48%) | 5 (3%) | 1 (20%) |
| NVP | 6 (3%) | 3 (50%) | 3 (9%) | 2 (67%) | 3 (2%) | 1 (33%) |
| DRV | 4 (2%) | 0 (0%) | 0 (0%) | - | 4 (3%) | 0 (0%) |
| EVG | 1 (1%) | 0 (0%) | 0 (0%) | - | 1 (1%) | 0 (0%) |
| RLP | 2 (1%) | 0 (0%) | 0 (0%) | - | 2 (1%) | 0 (0%) |
| **Age - per year increase** (median, [IQR]) | 10.5 [6.5 - 14.0] |  | 1.0 [0.4 – 1.7] |  | 11.6 [8.77 – 14.9] |  |
| 4 weeks - <3 years | 67 (18%) | 25 (37%) | 67 (100%) | 25 (37%) | 0 (0%) | - |
| 3 years - <6 years | 17 (4%) | 4 (24%) | 0 (0%) | - | 17 (5%) | 4 (24%) |
| 6 - <12 years | 154 (40%) | 29 (19%) | 0 (0%) | - | 154 (49%) | 29 (19%) |
| 12 - <18 years | 143 (38%) | 17 (12%) | 0 (0%) | - | 143 (45%) | 17 (12%) |
| **Weight - per kilo increase** (median, [IQR]) | 26.4 [18.4 - 41.0] |  | 7.0 [5.1 – 9.5] |  | 29.6 [22.9 - 44.8] |  |
| 3 - <6kg | 23 (6%) | 13 (57%) | 23 (34%) | 13 (57%) | 0 (0%) | - |
| 6 - <10kg | 35 (9%) | 11 (31%) | 34 (51%) | 10 (29%) | 1 (<1%) | 1 (100%) |
| 10 - <14kg | 14 (4%) | 2 (14%) | 9 (13%) | 1 (11%) | 5 (2%) | 1 (20%) |
| 14 - <20kg | 38 (10%) | 10 (26%) | 1 (1%) | 1 (100%) | 37 (12%) | 9 (24%) |
| 20 - <40kg | 173 (45%) | 30 (17%) | 0 (0%) | - | 173 (55%) | 30 (17%) |
| ≥40kg | 98 (26%) | 9 (9%) | 0 (0%) | - | 98 (31%) | 9 (9%) |
| **Middle-upper arm circumference - per cm increase** (median, [IQR]) (n=372) | 18.0 [15.5 - 21.7] |  | 12.7 [11.0 – 14.0] |  | 18.8 [16.5 - 22.5] |  |
| <15 | 72 (19%) | 29 (40%) | 54 (86%) | 23 (43%) | 18 (6%) | 6 (33%) |
| 15 - <18 | 107 (29%) | 23 (22%) | 9 (14%) | 2 (22%) | 98 (32%) | 21 (21%) |
| 18 - <21 | 79 (21%) | 11 (14%) | 0 (0%) | - | 79 (26%) | 11 (14%) |
| ≥21 | 114 (31%) | 12 (11%) | 0 (0%) | - | 114 (37%) | 12 (11%) |
| **Height-for-age z-score – per unit increase** (median, [IQR]) | -1.3 [-2.3 - -0.5] |  | -2.5 [-3.6 - -1.3] |  | -1.2 [-2 - -0.4] |  |
| <-3 | 52 (14%) | 19 (37%) | 26 (39%) | 11 (42%) | 26 (8%) | 8 (31%) |
| -3 - <-2 | 64 (17%) | 16 (25%) | 14 (21%) | 8 (57%) | 50 (16%) | 8 (16%) |
| -2 - <0 | 221 (58%) | 34 (15%) | 22 (33%) | 4 (18%) | 199 (63%) | 30 (15%) |
| ≥0 | 44 (12%) | 6 (14%) | 5 (7%) | 2 (40%) | 39 (12%) | 4 (10%) |
| **BMI-for-age z-score – per unit increase** (median, [IQR]) | -0.58 [-1.48 - 0.25] |  | -1.1 [-2.1 - 0.2] |  | -0.5 [-1.3 - 0.3] |  |
| <-3 | 21 (6%) | 13 (62%) | 9 (13%) | 7 (78%) | 12 (4%) | 6 (50%) |
| -3 - <-2 | 35 (9%) | 11 (31%) | 10 (15%) | 6 (60%) | 25 (8%) | 5 (20%) |
| -2 - <0 | 205 (54%) | 33 (16%) | 26 (39%) | 7 (27%) | 179 (57%) | 26 (15%) |
| ≥0 | 120 (32%) | 18 (15%) | 22 (33%) | 5 (23%) | 98 (31%) | 13 (13%) |
| **CD4 percentage - per % increase** (median, [IQR]) (n=377) | 20 [12 - 28] |  | 24 [17 – 34] |  | 19 [10 - 27] |  |
| <15% | 125 (33%) | 31 (25%) | 11 (17%) | 10 (91%) | 144 (36%) | 21 (18%) |
| 15 - <25% | 126 (33%) | 25 (20%) | 26 (41%) | 9 (35%) | 100 (32%) | 16 (16%) |
| ≥25% | 126 (33%) | 17 (13%) | 27 (42%) | 4 (15%) | 99 (32%) | 13 (13%) |
| **CD4/CD8 ratio – per 0.1 increase** (median, [IQR]) (n=369) | 0.4 [0.2 - 0.7] |  | 0.5 [0.3 – 0.8] |  | 0.4 [0.2 – 0.7] |  |
| <0.2 | 89 (23%) | 26 (29%) | 6 (9%) | 6 (100%) | 83 (26%) | 20 (24%) |
| 0.2 - <0.6 | 165 (43%) | 28 (17%) | 30 (45%) | 12 (40%) | 135 (43%) | 16 (12%) |
| 0.6 - <1.0 | 78 (20%) | 12 (15%) | 14 (21%) | 2 (14%) | 64 (20%) | 10 (16%) |
| ≥1.0 | 49 (13%) | 9 (18%) | 17 (25%) | 5 (29%) | 32 (10%) | 4 (13%) |
| **Viral load – copies/mL** |  |  |  |  |  |  |
| <1,000 | 29 (8%) | 5 (17%) | 4 (6%) | 2 (50%) | 25 (8%) | 3 (12%) |
| 1,000 - <10,000 | 66 (18%) | 10 (15%) | 5 (8%) | 2 (40%) | 61 (19%) | 8 (13%) |
| 10,000 - <50,000 | 85 (23%) | 13 (15%) | 8 (13%) | 3 (38%) | 77 (25%) | 10 (13%) |
| 50,000 - <100,000 | 47 (13%) | 4 (9%) | 3 (5%) | 0 (0%) | 44 (14%) | 4 (9%) |
| 100,000 - <1,000,000 | 129 (34%) | 36 (28%) | 31 (50%) | 14 (45%) | 98 (31%) | 22 (22%) |
| ≥1,000,000 | 19 (5%) | 5 (26%) | 11 (18%) | 2 (18%) | 8 (3%) | 3 (38%) |
| (median, [IQR] – log10) (n=375) | 4.7 [4.0 – 5.3] |  | 5.4 [4.5 – 5.9] |  | 4.7 [3.9 – 5.2] |  |
| <100,000 | 227 (61%) | 32 (14%) | 20 (32%) | 7 (35%) | 207 (66%) | 25 (12%) |
| ≥100,000 | 148 (39%) | 41 (28%) | 42 (68%) | 16 (38%) | 106 (34%) | 25 (24%) |
| **Ongoing WHO stage 3/4 event (No)** | 308 (81%) | 50 (16%) | 48 (72%) | 14 (29%) | 260 (83%) | 36 (14%) |
| Yes | 73 (19%) | 25 (34%) | 19 (28%) | 11 (58%) | 54 (17%) | 14 (26%) |
| **Haemoglobin - per 1 unit increase (g/dL)** (median, [IQR]) (n=379) | 11.2 [10.0 - 12.5] |  | 9.4 [8.7 – 10.3] |  | 11.6 [10.5 – 12.8] |  |
| <10 | 89 (23%) | 24 (27%) | 40 (62%) | 13 (33%) | 49 (16%) | 11 (22%) |
| 10 - <12 | 162 (43%) | 36 (22%) | 24 (37%) | 10 (42%) | 138 (44%) | 26 (19%) |
| ≥12 | 128 (34%) | 13 (10%) | 1 (2%) | 0 (0%) | 127 (40%) | 13 (10%) |
| **Platelets - per 10 units increase (10³/µL)** (median, [IQR]) (n=379) | 325 [252 - 407] |  | 387 [282 – 523] |  | 316 [250 – 391] |  |
| <250 | 91 (24%) | 22 (24%) | 13 (20%) | 8 (62%) | 78 (25%) | 14 (18%) |
| 250 - <325 | 97 (26%) | 16 (16%) | 8 (12%) | 1 (13%) | 89 (28%) | 15 (17%) |
| 325 - <400 | 90 (24%) | 12 (13%) | 17 (26%) | 5 (29%) | 73 (23%) | 7 (10%) |
| ≥400 | 101 (27%) | 23 (23%) | 27 (42%) | 9 (33%) | 74 (24%) | 14 (19%) |
| **Neutrophils - per 1 unit increase (10⁹/L)** (median, [IQR]) (n=358) | 2.1 [1.5 - 3.0] |  | 2.5 [1.7 – 3.7] |  | 2.0 [1.4 – 2.9] |  |
| <1 | 29 (8%) | 5 (17%) | 6 (9%) | 2 (33%) | 23 (8%) | 3 (13%) |
| 1 - <2 | 140 (39%) | 24 (17%) | 15 (23%) | 4 (27%) | 125 (43%) | 20 (16%) |
| 2 - <3 | 96 (27%) | 15 (16%) | 23 (35%) | 8 (35%) | 73 (25%) | 7 (10%) |
| ≥3 | 93 (26%) | 27 (29%) | 21 (32%) | 9 (43%) | 72 (25%) | 18 (25%) |
| **Sex – Male** | 181 (48%) | 39 (22%) | 33 (49%) | 13 (39%) | 148 (47%) | 26 (18%) |
| Female | 200 (52%) | 36 (18%) | 34 (51%) | 12 (35%) | 166 (53%) | 24 (14%) |
| **Primary caregiver - Female** (n=321) | 270 (84%) | 59 (22%) | 56 (97%) | 23 (41%) | 214 (81%) | 36 (17%) |
| Male | 51 (16%) | 6 (12%) | 2 (3%) | 0 (0%) | 49 (19%) | 6 (12%) |
| **Region (non-African site)** | 68 (18%) | 5 (7%) | 0 (0%) | - | 68 (22%) | 5 (7%) |
| African site | 313 (82%) | 70 (22%) | 67 (100%) | 25 (37%) | 246 (78%) | 45 (18%) |

Abbreviations: BMI, body mass index; DRV, darunavir; DTG, dolutegravir; EFV, efavirenz; EVG, elvitegravir; IQR, interquartile range; LOP, lopinavir; NVP, nevirapine; RLP, rilpivirine; SOC, standard of care; WHO, World Health Organisation.

**Appendix 2:** Risk of treatment failure by 96 weeks by age at ART initiation in model adjusting for trial arm only and final multivariable model.

Panel A: univariable model adjusted for trial arm only (Table 1). Panel B: final multivariable model (without interaction terms) (Table 2). Shaded areas indicate 95% confidence intervals.

**Appendix 3:** Distribution of variable selection in domain-specific and full multivariable models based on 1,000 bootstrap samples.

Bootstrap models included trial arm and log_e_(age) and tested other factors independently (with no interactions). Black squares denote selected variables. Count is the number of bootstrap samples each model was selected in.

**Appendix 4:** Final multivariable model-based hazard ratios for treatment failure by age and CD4%

Hazard ratios are expressed relative to a reference child aged 10 years with a CD4 percentage of 20%, and based on the final multivariable model (Table 3).

**Appendix 5:** Final multivariable model fitted within each trial arm.

|  |  |  | **Final Model** | | | **Final Model - within trial arm** | | | | | | |
| --- | --- | --- | --- | --- | --- | --- | --- | --- | --- | --- | --- | --- |
|  |  |  | **(N=381)** | | | **DTG**  **(N=189)** | | | **SOC**  **(N=192)** | | | **P (for interaction with trial arm)** |
| **Factor** | **Comparison** | **Assumption** | **HR** | **95% CI** | **p-value** | **HR** | **95% CI** | **p-value** | **HR** | **95% CI** | **p-value** |  |
| Trial Arm | DTG vs SOC |  | 0.47 | (0.29-0.77) | 0.002 |  |  |  |  |  |  |  |
| Age (yr) | 1 vs 10 | CD4% = 20% | 2.40 | (1.58-3.65) | <0.001 | 2.77 | (1.29-5.95) | 0.01 | 2.32 | (1.39-3.89) | 0.002 | 0.67 |
|  | 2 vs 10 | CD4% = 20% | 1.84 | (1.38-2.47) |  | 2.04 | (1.19-3.48) |  | 1.80 | (1.26-2.59) |  |  |
|  | 5 vs 10 | CD4% = 20% | 1.30 | (1.15-1.48) |  | 1.36 | (1.08-1.71) |  | 1.29 | (1.10-1.51) |  |  |
|  | 18 vs 10 | CD4% = 20% | 0.80 | (0.72-0.89) |  | 0.77 | (0.63-0.94) |  | 0.81 | (0.71-0.92) |  |  |
| BMI-for-age | per unit increase |  | 0.82 | (0.70-0.96) | 0.01 | 0.79 | (0.55-1.14) | 0.21 | 0.84 | (0.70-1.00) | 0.05 | 0.28 |
| CD4 percentage | 15% vs 20% | Age = 1 | 1.39 | (1.16-1.66) | 0.002 | 1.46 | (1.05-2.03) | 0.04 | 1.36 | (1.09-1.69) | 0.03 | 0.16 |
|  | 30% vs 20% | Age = 1 | 0.52 | (0.36-0.75) |  | 0.47 | (0.24-0.90) |  | 0.55 | (0.35-0.84) |  |  |
|  | 15% vs 20% | Age = 10 | 1.07 | (0.94-1.20) |  | 1.22 | (0.94-1.58) |  | 1.01 | (0.87-1.16) |  |  |
|  | 30% vs 20% | Age = 10 | 0.88 | (0.69-1.13) |  | 0.68 | (0.40-1.14) |  | 0.99 | (0.74-1.32) |  |  |
| Region | African site vs non-African site |  | 2.09 | (0.82-5.31) | 0.09 | 3.14 | (0.41-24.2) | 0.20 | 1.72 | (0.60-4.98) | 0.29 | 0.55 |

Full multivariable model selected in both trial arms was run separately in both DTG and SOC groups (with no selection of variables). All p-values are from likelihood ratio tests. Interaction p-values were obtained by adding treatment-covariate interaction terms individually to the final multivariable model.

Abbreviations: BMI, body mass index; CI, confidence interval; DTG, dolutegravir; HR, hazard ratio; SOC, standard of care.

**Appendix 6:** Final multivariable model when region was excluded from model selection

| **Factor** | **Comparison** | **Assumption** | **HR** | **95% CI** | **p-value** |
| --- | --- | --- | --- | --- | --- |
| Trial arm | DTG vs SOC |  | 0.47 | (0.29-0.77) | 0.002 |
| Age (yr) | 1 vs 10 | CD4% = 20% | 2.64 | (1.76-3.95) | <0.001 |
|  | 2 vs 10 | CD4% = 20% | 1.97 | (1.49-2.61) |  |
|  | 5 vs 10 | CD4% = 20% | 1.34 | (1.19-1.51) |  |
|  | 18 vs 10 | CD4% = 20% | 0.78 | (0.70-0.87) |  |
| BMI-for-age | per unit increase |  | 0.82 | (0.70-0.96) | 0.01 |
| CD4 percentage | 15% vs 20% | Age = 1 | 1.39 | (1.16-1.67) | 0.002 |
|  | 30% vs 20% | Age = 1 | 0.52 | (0.36-0.74) |  |
|  | 15% vs 20% | Age = 10 | 1.06 | (0.94-1.20) |  |
|  | 30% vs 20% | Age = 10 | 0.89 | (0.69-1.14) |  |

P-values are from likelihood tests. For age and CD4%, p-values were obtained from joint likelihood ratio tests of the main effect and the age x CD4% interaction term.

Abbreviations: BMI, body mass index; CI, confidence interval; DTG, dolutegravir; HR, hazard ratio; WHO, World Health Organisation.

**Appendix 7:** Risk of treatment failure by age, BMI-for-age and CD4 percentage at ART initiation in the SOC arm

Risks are predicted from the full multivariable model and shown **(a)** by age at ART initiation (1, 2, 5, and 18 years), with CD4% and BMI-for-age Z-score fixed at their medians (20% and -0.58 Z-score, respectively); **(b)** by CD4% at ART initiation (15% and 30%), with age fixed at 1yr and 10yr and BMI-for-age Z-score fixed at its median; **(c)** by BMI-for-age Z-score at ART initiation (−3, −2, 0, and 1), with age fixed at 10yr and CD4% fixed at its median (20%). Panel **(d)** shows the comparison of predicted high-risk (Age = 1, CD4% = 20%, BMI-for-age Z-score = -2) versus low-risk (Age = 10, CD4% = 30%, BMI-for-age Z-score = 1) profiles based on baseline characteristics, with all predictors set as specified. All predictions assume treatment with SOC at an African study site. Results are presented as probabilities of treatment failure by 96 weeks, with 95% confidence intervals.

Abbreviations: ART, antiretroviral therapy; BMI, body mass index; CI, confidence interval; SOC, standard-of-care.

**Appendix 8:** Full multivariable model using a higher threshold for virological failure (confirmed viral load ≥1000 copies/mL by 96 weeks) within the composite endpoint.

|  |  |  | **Selected Full Multivariable Model** | | | **Alternative Full Multivariable Model 1** | | | **Alternative Full Multivariable Model 2** | | |
| --- | --- | --- | --- | --- | --- | --- | --- | --- | --- | --- | --- |
| **Factor** | **Comparison** | **Assumption** | **HR** | **95% CI** | **p-value** | **HR** | **95% CI** | **p-value** | **HR** | **95% CI** | **p-value** |
| Trial arm | DTG vs SOC |  | 0.45 | (0.27-0.76) | 0.002 | 0.47 | (0.28-0.78) | 0.003 | 0.46 | (0.28-0.78) | 0.003 |
| Age – per year increase | 1 vs 10 | CD4% = 20% | 2.41 | (1.52-3.84) | <0.001 | 2.62 | (1.69-4.06) | <0.001 | 2.44 | (1.57-3.80) | <0.001 |
|  | 2 vs 10 | CD4% = 20% | 1.85 | (1.34-2.56) |  | 1.96 | (1.44-2.66) |  | 1.87 | (1.37-2.54) |  |
|  | 5 vs 10 | CD4% = 20% | 1.30 | (1.13-1.50) |  | 1.34 | (1.17-1.53) |  | 1.31 | (1.15-1.49) |  |
|  | 18 vs 10 | CD4% = 20% | 0.80 | (0.71-0.90) |  | 0.78 | (0.70-0.88) |  | 0.80 | (0.71-0.89) |  |
| BMI-for-age | per unit increase |  |  |  |  | 0.90 | (0.75-1.08) | 0.27 | 0.84 | (0.71-1.00) | 0.05 |
| Height-for-age | per unit increase |  | 0.85 | (0.70-1.03) | 0.10 |  |  |  |  |  |  |
| CD4 percentage | 15% vs 20% | Age = 1 | 1.31 | (1.09-1.57) | 0.01 | 1.31 | (1.09-1.58) | 0.02 | 1.33 | (1.11-1.61) | 0.008 |
|  | 30% vs 20% | Age = 1 | 0.58 | (0.41-0.84) |  | 0.58 | (0.40-0.85) |  | 0.56 | (0.39-0.82) |  |
|  | 15% vs 20% | Age = 10 | 1.08 | (0.94-1.24) |  | 1.08 | (0.95-1.24) |  | 1.10 | (0.96-1.26) |  |
|  | 30% vs 20% | Age = 10 | 0.86 | (0.66-1.12) |  | 0.85 | (0.65-1.11) |  | 0.83 | (0.64-1.08) |  |
| Ongoing WHO 3/4 event | Yes vs No |  | 1.80 | (1.06-3.08) | 0.03 | 1.73 | (0.96-3.10) | 0.07 |  |  |  |
| Region (African site) | African site vs non-African site |  |  |  |  |  |  |  | 1.81 | (0.71-4.65) | 0.19 |

Of the 56 participants who met the original virological criterion (≥400 copies/mL), 9 did not meet the revised ≥1000 copies/mL definition and were censored accordingly. No participants switched treatment or met another component of the primary endpoint between meeting the original and revised virological endpoints.

Empty cells denote variables that were considered but not retained in the model selection procedure (backwards elimination (p<0.1)). P-values are from likelihood tests.

Alternative full multivariable model 1 shows the selected full multivariable model but including BMI-for-age rather than height-for-age using the revised definition for failure. This model showed an equally good fit compared to the selected full multivariable model (∆AIC=1).

Alternative full multivariable model 2 represents the final multivariable model covariates (table 3) fitted to the dataset using the revised definition for failure. This model showed a good fit but was marginally poorer than the selected full multivariable model (column 1, this table) (∆AIC=3).

Abbreviations: BMI, body mass index; CI, confidence interval; DTG, dolutegravir; HR, hazard ratio; WHO, World Health Organisation.

**Appendix 9:** Associations between treatment failure and final model predictors within age groups

|  | **Full Model - within age group** | | | | | | |  | |  | |  |
| --- | --- | --- | --- | --- | --- | --- | --- | --- | --- | --- | --- | --- |
|  | **<3yrs**  **(N=67)** | | | **≥3yrs**  **(N=314)** | | | **P (for interaction with age group)** | **Alternative Model (≥3yrs)** | | | | |
| **Factor** | **HR** | **95% CI** | **p-value** | **HR** | **95% CI** | **p-value** |  | **HR** | **95% CI** | | **p-value** | |
| Trial Arm – DTG | 0.62 | (0.27-1.42) | 0.25 | 0.41 | (0.22-0.75) | 0.002 | 0.62 | 0.39 | (0.21-0.72) | | 0.002 | |
| Age – per year increase | 0.58 | (0.30-1.12) | 0.09 | 0.95 | (0.88-1.03) | 0.22 | 0.67 | 0.96 | (0.89-1.04) | | 0.34 | |
| BMI-for-age – per unit increase | 0.84 | (0.67-1.06) | 0.14 | 0.79 | (0.62-1.01) | 0.06 | 0.44 | 0.83 | (0.66-1.04) | | 0.10 | |
| CD4 percentage – per % increase | 0.93 | (0.89-0.97) | 0.002 | 0.99 | (0.96-1.02) | 0.41 | 0.33 |  |  | |  | |
| Viral load (≥100,000 copies/mL) |  |  |  |  |  |  |  | 2.06 | (1.15-3.68) | | 0.02 | |
| Region - African site | - | - | - | 2.08 | (0.79-5.48) | 0.11 |  | 2.31 | (0.87-6.11) | | 0.07 | |

All predictors here were included as linear terms without interaction to illustrate associations within each age group. All participants <3yrs were at an African site. The alternative model (≥3yrs) demonstrates the full multivariable model selected when the dataset was restricted to those ≥3yrs only, keeping age linear. P-values are from likelihood tests.

Abbreviations: BMI, body mass index; CI, confidence interval; DTG, dolutegravir; HR, hazard ratio; SOC, standard of care.

**Appendix 10**: Alternative full multivariable models under restrictions to availability of laboratory measurements.

|  | **Scenario 1** | | | **Scenario 2** | | |
| --- | --- | --- | --- | --- | --- | --- |
| **Factor** | **HR** | **95% CI** | **p-value** | **HR** | **95% CI** | **p-value** |
| Trial arm - DTG | 0.45 | (0.27-0.73) | <0.001 | 0.46 | (0.28-0.75) | 0.001 |
| Age – per year increase | * | * | 0.009 | * | * | <0.001 |
| BMI-for-age – per unit increase | 0.76 | (0.65-0.89) | <0.001 | 0.80 | (0.68-0.95) | 0.01 |
| Viral Load (≥100,000 copies/mL) | 1.62 | (1.01-2.61) | 0.05 |  |  |  |
| Ongoing WHO 3/4 event (Yes) |  |  |  | 1.70 | (0.99-2.92) | 0.06 |
| Region (African site) | 2.31 | (0.91-5.88) | 0.05 |  |  |  |

To evaluate the impact of restrictions to the availability of laboratory measurements (CD4%, viral load, haemoglobin, platelets, neutrophils) on model selection, the same selection process as in the main analysis was repeated under two alternative scenarios:

Scenario 1: CD4% unavailable
Scenario 2: CD4% and viral load unavailable

*Age was transformed using log_e_(age). P-values are from likelihood tests.
